# Maternal Loneliness and Technoference During Early Childhood: The Mediating Roles of Nomophobia and Social Media Addiction

**DOI:** 10.3390/ejihpe16090132

**Published:** 2026-09-02

**Authors:** Laura Elvira Prino, Donatella Scarzello

**Affiliations:** Department of Philosophy and Education Sciences, University of Turin, 10124 Turin, Italy; donatella.scarzello@unito.it

**Keywords:** technoference, early childhood, loneliness, nomophobia, social media addiction, mother–child interactions

## Abstract

Maternal technoference has increasingly been recognized as a factor that may compromise mothers’ emotional availability and the quality of mother–child interactions, particularly during early childhood, when responsive caregiving and co-regulation play a pivotal role in children’s development. However, the psychological mechanisms underlying this phenomenon remain poorly understood. Maternal loneliness may represent an important vulnerability factor, although the processes linking loneliness to technoference have yet to be clarified. This study examined whether nomophobia and social media addiction mediate the association between maternal loneliness and technoference. This cross-sectional study included 355 Italian mothers of children aged 0–6 years, who completed measures of loneliness (UCLA 3-item Loneliness Scale), nomophobia (NMP-Q), social media addiction (BSMAS), and technoference (DISRUPT). A parallel mediation model was tested while controlling for maternal age. Loneliness was positively associated with both nomophobia and social media addiction, which were, in turn, positively associated with technoference. Both indirect effects were significant, whereas the direct association between loneliness and technoference was no longer significant. These findings suggest that maternal loneliness may be associated with technoference through its link with nomophobia and social media addiction, highlighting the importance of understanding technoference as a relational phenomenon rather than merely a consequence of technology use.

## 1. Introduction

Early childhood is a particularly sensitive developmental period during which relational experiences play a fundamental role in shaping children’s socioemotional, cognitive, and neurobiological development (Bowlby, 1969; Sroufe, 2005). During this developmental period, children’s self-regulatory capacities are still immature, and they rely heavily on caregivers to regulate emotional states, organize behavior, and make sense of their experiences. Through daily reciprocal and contingent interactions characterized by ongoing processes of co-regulation, caregivers gradually foster the development of emotion regulation, social competence, and trust in relationships, laying the foundations for later psychological adjustment (Bornstein, 2019; Feldman, 2007).

Parental sensitivity is among the primary determinants of early caregiving quality and is defined as the caregiver’s ability to accurately perceive, interpret, and respond promptly and appropriately to the child’s cues (Ainsworth et al., 1978; Bornstein, 2019). Parental sensitivity provides the foundation for responsive caregiving and promotes the development of secure attachment, supporting children’s emotional, cognitive, linguistic, and social development (Bornstein, 2019; Landry et al., 2006; Leerkes, 2010). These processes unfold through ongoing co-regulation, in which caregivers and children mutually regulate attention, emotions, and behavior. This affective synchrony is considered one of the primary mechanisms through which children gradually internalize increasingly autonomous emotion regulation strategies (Feldman, 2007). Consistent with this perspective, the still-face paradigm has shown that even brief disruptions in reciprocal interaction rapidly alter infants’ emotion regulation, underscoring the importance of caregivers’ emotional availability for children’s healthy development (Tronick, 2007).

Although both parents contribute substantially to children’s development, research on early caregiving has focused predominantly on mothers, who continue to be the primary caregivers during early childhood in most cultural contexts and remain more involved in children’s daily care and emotion regulation (Bornstein, 2019; Kan et al., 2011; Moreno-Colom, 2017). Consequently, identifying the factors that may compromise maternal emotional availability in everyday interactions is particularly important during early childhood, when children rely heavily on caregivers’ sensitivity and responsiveness to regulate their internal states and develop internal working models of relationships (Bowlby, 1969; Sroufe, 2005).

Maternal emotional availability extends beyond the mother’s mere physical presence and represents a multidimensional relational construct, of which sensitivity is one component. It also encompasses the ability to appropriately structure interactions, maintain a nonintrusive and nonhostile stance, and foster emotionally reciprocal exchanges with the child (Biringen, 2000; Easterbrooks & Biringen, 2000). Consequently, any factor that interferes with these dimensions may compromise the quality of everyday interactions and limit opportunities for co-regulation, with potential implications for children’s development (Feldman, 2007; Tronick, 2007).

In recent years, the widespread use of digital technologies has profoundly changed how parents experience family relationships and manage everyday life. Mobile devices offer valuable opportunities to maintain social connections, access information, and obtain parenting-related resources, but their constant availability also creates ongoing competition for caregivers’ attentional resources (McDaniel, 2019). According to McDaniel (2019), distraction caused by digital devices may compromise each of the components of parental sensitivity described by Ainsworth by reducing caregivers’ ability to accurately perceive children’s cues, interpret them appropriately, and respond in a prompt, contingent, and appropriate manner. McDaniel (2019) identifies three main mechanisms through which this occurs. These mechanisms include displacement (time spent on devices replacing parent–child interactions), divided attention (reduced responsiveness due to multitasking), and emotional engagement with digital content, which may diminish caregivers’ emotional availability during interactions with their child (McDaniel, 2019). Importantly, the primary concern is not device use itself, but the extent to which it compromises caregivers’ emotional availability during everyday interactions.

This form of interference has been conceptualized as technoference, defined as the intrusion of digital technologies into everyday interpersonal interactions (McDaniel & Radesky, 2018). Within the family context, technoference occurs when caregivers repeatedly shift their attention from the child to digital devices, disrupting the reciprocal processes that underpin early caregiving relationships. A concept similar to “technoference” is “phubbing”, which is defined as the act of snubbing or ignoring an interlocutor in a social setting by directing attention to one’s smartphone instead of engaging in face-to-face interaction (Karadağ et al., 2015; Maftei & Măirean, 2024). Despite inconsistencies in how these constructs are defined and differentiated in the literature (Frackowiak et al., 2024), they substantially overlap in their processes and effects within the family context.

Evidence accumulated over the past decade indicates that technoference is associated with reduced joint attention, eye contact, verbal interactions, emotional reciprocity, and the quality of shared play, as well as with lower parental sensitivity and responsiveness and poorer parent–child relationship quality (Abels et al., 2018; Braune-Krickau et al., 2021; McDaniel, 2019). Essentially, digital distractions shift the dyadic dynamic toward a state of “absent presence,” leaving the parent physically proximate yet psychologically unavailable (Gergen, 2002). Recent reviews have further linked technoference to adverse child outcomes, such as higher internalizing and externalizing problems and lower social competence (McDaniel, 2019; McDaniel & Radesky, 2018; Toledo-Vargas et al., 2025).

Although the consequences of technoference are now well established, far less is known about the psychological processes that make some parents more prone to engaging in technology-related disruptions during interactions with their children. Smartphones have become an integral part of everyday family life, yet parents differ considerably in the extent to which they allow digital devices to interfere with caregiving interactions (McDaniel, 2019; McDaniel & Radesky, 2018). This variability suggests that the widespread availability of digital technologies alone is insufficient to explain technoference and that specific individual and relational characteristics may heighten susceptibility to digital distractions. Recent studies have identified problematic engagement with digital technologies, social media addiction, and nomophobia (defined as the discomfort or anxiety associated with being unable to access or use one’s smartphone; Bhattacharya et al., 2019; Bragazzi & Del Puente, 2014) as potential psychological antecedents of technoference (Dolev-Cohen & Ricon, 2024; Karaduman et al., 2024; McDaniel & Radesky, 2018; Sun & Miller, 2023).

Recent research suggests that some parents’ vulnerability to technoference may be explained by emotional and relational experiences that promote compensatory engagement with digital technologies (Dolev-Cohen & Ricon, 2024; McDaniel, 2019; Sun & Miller, 2023). Among these, loneliness has emerged as a relevant factor because it is one of the main antecedents of compensatory engagement with digital technologies and related problematic behaviors, including nomophobia and phubbing (Heng et al., 2023; Hu & Xiang, 2024; Maftei & Măirean, 2024; Moretta & Buodo, 2020).

Loneliness is defined as the perceived discrepancy between desired and actual social relationships (D. W. Russell, 1996; D. Russell et al., 1978), is characterized as a psychological state stemming from an unmet need for meaningful interactions (de Jong-Gierveld, 1987), and is distinct from quantifiable dimensions of interpersonal contact, such as social network size (Van Baarsen et al., 2001). Loneliness is now recognized as a major public health concern (Constantinidou et al., 2025; Nowland et al., 2018). The transition to parenthood is a particularly vulnerable period, with approximately one-third of parents experiencing persistently high levels of loneliness, especially mothers of preschool-aged children (Nowland et al., 2021). Reorganization of social networks, increased caregiving responsibilities, and fewer opportunities for social interaction may contribute to loneliness, even when an apparently adequate support system is available (Nowland et al., 2021, 2024). Parental loneliness has also been associated with poorer parental well-being and less favorable developmental outcomes for children, highlighting it as a significant vulnerability factor within early caregiving relationships (Nowland et al., 2024).

The Compensatory Internet Use Theory posits that individuals may engage with digital technologies to compensate for unmet psychological needs or to regulate negative emotional states (Kardefelt-Winther, 2014). In line with this perspective, major theoretical models of problematic Internet use suggest that pre-existing psychological vulnerabilities may foster maladaptive engagement with digital technologies as a strategy for emotion regulation (Brand et al., 2019; Caplan, 2010; Davis, 2001; Moretta & Buodo, 2020). Similarly, Nowland et al. (2018) proposed a dynamic framework in which the relationship between loneliness and technology use depends on how digital technologies are used. Specifically, technology use may help alleviate loneliness when it facilitates meaningful social connections, but it may exacerbate loneliness when it replaces offline interactions or is used to avoid relational distress. Moreover, digitally mediated interactions may also exacerbate loneliness when they fail to satisfy individuals’ need for belonging or when they foster unfavorable social comparisons (Dibb & Foster, 2021; Hall et al., 2023).

Empirical evidence supports this theoretical framework. Reviews, meta-analyses, and longitudinal studies consistently identify loneliness as one of the main antecedents of problematic engagement with digital technologies (Hu & Xiang, 2024; Kim, 2017; Moretta & Buodo, 2020; Zhang et al., 2020; Zhao et al., 2024). Loneliness has also been associated with nomophobia (Constantinidou et al., 2025; Dinç et al., 2025; Heng et al., 2023; Zwilling, 2022), problematic Internet use, and social media addiction (MacDonald & Schermer, 2021; Mitropoulou, 2024; Orsolini et al., 2023). Notably, Kim (2017) found that loneliness predicted problematic smartphone use through an escape motivation that drives lonely individuals toward smartphone-mediated communication rather than face-to-face interaction, providing empirical support for the compensatory mechanism posited by the Compensatory Internet Use Theory. Furthermore, growing evidence suggests that loneliness is associated with phubbing in relationships between adults (Furmańska et al., 2025; Maftei & Măirean, 2024; Zhan et al., 2022). Given the close conceptual overlap between phubbing and technoference within the family context (Maftei & Măirean, 2024), it is plausible that similar mechanisms contribute to technology-related disruptions of mother–child interactions. However, although loneliness has been consistently associated with problematic engagement with digital technologies and phubbing, no previous study has directly examined whether these mechanisms may explain the association between maternal loneliness and technoference during early childhood.

Taken together, these findings suggest that technoference should be understood not merely as a consequence of the widespread use of digital technologies, but as the behavioral expression of broader emotional and relational regulatory processes. However, research on loneliness has primarily focused on its role as an antecedent of problematic engagement with digital technologies, whereas research on technoference has largely examined its consequences for parent–child interactions rather than the psychological processes that may contribute to its occurrence. Moreover, despite the growing interest in parental loneliness, few studies have examined these constructs together within mother–child relationships during early childhood (Nowland et al., 2021, 2024). Consequently, the nature of the association between maternal loneliness and technoference remains insufficiently understood.

### Current Study

The present study aimed to examine the association between maternal loneliness and technoference during interactions with children aged 0 to 6 years, considering the potential indirect roles of nomophobia and social media addiction. Based on the existing literature and the Compensatory Internet Use Theory (Kardefelt-Winther, 2014), we formulated the following hypotheses:

**H1.**  *Higher levels of maternal loneliness would be associated with higher levels of nomophobia and social media addiction*.

**H2.**  *Higher levels of nomophobia and social media addiction would be associated with higher levels of technoference during mother–child interactions*.

**H3.**  *Maternal loneliness would be indirectly associated with technoference through nomophobia and social media addiction*.

## 2. Materials and Methods

### 2.1. Participants and Procedure

Participants were 355 mothers of children from birth to 6 years of age residing in Italy. They were recruited through a snowball sampling strategy and completed an online questionnaire administered via Google Forms. Data were collected anonymously, participation was voluntary and uncompensated, and all participants provided informed consent prior to participation. The study was conducted in accordance with the ethical principles of the American Psychological Association (APA) and the Declaration of Helsinki and received ethical approval from the Ethics Committee of the University of Turin.

Eligible participants were mothers of children aged 0–6 years. Mothers were included in the analysis if they had less than 5% missing responses across the study measures. Mothers whose target child was older than 6 years and those with 5% or more missing responses were excluded from the analysis. A total of 373 mothers initially participated in the study. Of these, 18 were excluded because they did not meet the inclusion criteria, resulting in a final analytic sample of 355 mothers. Data were collected between February 2024 and September 2024.

Mothers’ ages ranged from 21 to 51 years (M = 33.55, SD = 4.58). The target children ranged from 0 to 6 years of age (M = 2.15, SD = 1.87), with 45.4% boys and 54.6% girls. Most mothers were married (54.4%) or cohabiting (42.3%), and the majority held a university degree (59.7%).

### 2.2. Measures

Maternal technoference was assessed using the *Distraction In Social Relations and Use of Parent Technology* scale (DISRUPT, McDaniel, 2021), a 4-item questionnaire designed to capture parents’ tendencies toward problematic smartphone use during time spent with their child. Items are rated on a 6-point Likert scale ranging from 1 (*Strongly disagree*) to 6 (*Strongly agree*), with higher scores indicating greater technoference. The DISRUPT demonstrated good reliability and evidence of convergent, divergent, and predictive validity (McDaniel, 2021). As no validated Italian version of the DISRUPT was available, the scale was translated into Italian and then back-translated into English following established guidelines for the cross-cultural adaptation of assessment instruments (van de Vijver & Hambleton, 1996). The back-translated version was compared with the original to ensure linguistic and conceptual equivalence. Internal consistency in the present sample was good (Cronbach’s α = 0.86, McDonald’s ω = 0.86).

Loneliness was assessed using the UCLA 3-item Loneliness Scale (Hughes et al., 2004), an abbreviated version of the UCLA Loneliness Scale (D. W. Russell, 1996). The scale consists of 3 items assessing subjective feelings of social isolation and dissatisfaction with one’s social relationships (*“How often do you feel that you lack companionship?”, “How often do you feel left out?”, “How often do you feel isolated from others?”*). Participants rated each item on a 3-point Likert scale ranging from 1 (*Never*) to 3 (*Often*), with higher scores indicating greater perceived loneliness. The UCLA 3-item Loneliness Scale has demonstrated adequate reliability and good convergent, discriminant, and known-groups validity, supporting its use as a brief dimensional measure of loneliness (Hughes et al., 2004; Gosling et al., 2024). The scale demonstrated good internal consistency (Cronbach’s α = 0.85, McDonald’s ω = 0.86).

Nomophobia was assessed using the Nomophobia Questionnaire (NMP-Q; Yildirim & Correia, 2015; Italian version by Adawi et al., 2018). The questionnaire comprises 20 items assessing four dimensions of nomophobia—not being able to access information, giving up convenience, not being able to communicate, and losing connectedness. Responses are provided on a 7-point Likert scale ranging from 1 *(Strongly disagree) to 7 (Strongly agree)*, with higher scores reflecting greater nomophobia. Previous studies have provided evidence for the reliability and structural validity of the NMP-Q across different language versions, including its Italian adaptation (Adawi et al., 2018; Jahrami et al., 2023). In the present sample, the scale showed excellent internal consistency (Cronbach’s α = 0.93, McDonald’s ω = 0.93). A total score was computed and used in the analyses.

Problematic social media use was assessed using the Bergen Social Media Addiction Scale (BSMAS; Andreassen et al., 2017; Italian version by Monacis et al., 2017). The scale includes six items reflecting the core components of behavioral addiction applied to social media use which has consistently demonstrated a unidimensional factor structure. Items are rated on a 5-point Likert scale ranging from 1 (*Very rarely*) to 5 (*Very often*), with higher scores indicating greater levels of social media addiction. Evidence from different populations supports the reliability and unidimensional structure of the BSMAS, with satisfactory psychometric properties also reported for the Italian version (Monacis et al., 2017; Bottaro et al., 2026). Internal consistency in the present study was acceptable (Cronbach’s α = 0.77, McDonald’s ω = 0.76).

### 2.3. Statistical Analysis

Data were analyzed using IBM SPSS Statistics (Version 30). Given that all study variables were assessed using self-report measures completed by the same respondents at a single time point, Harman’s single-factor test was conducted to examine the potential influence of common method variance. The first unrotated factor accounted for 33.61% of the total variance, suggesting that common method bias was unlikely to represent a substantial concern in the present study. After conducting descriptive analyses, preliminary analyses were performed to check data distribution and ensure that assumptions for parametric analyses were met. Analyses of variance (ANOVA) and bivariate Pearson’s correlations were performed to examine associations among the study variables, depending on their level of measurement. To test the research hypotheses, a parallel mediation analysis was performed using PROCESS macro version 4.2 (Model 4; Hayes, 2022). Although the overall sample comprised *N* = 355 mothers, the mediation analysis was based on *n* = 343 complete cases because of missing data on the nomophobia measure; no missing data were observed for the other study variable. This effective sample size was considered adequate based on simulation studies indicating that approximately 162 participants are sufficient to achieve 0.80 power for detecting indirect effects with small-to-medium component paths using percentile bootstrap methods (Fritz & MacKinnon, 2007). The significance of the indirect effects was assessed using percentile bootstrap 95% confidence intervals (CI) based on 5000 resamples. Indirect effects were considered statistically significant when the 95% CI did not include zero. For all other analyses, the level of statistical significance was set at *p* < 0.05, two-tailed.

## 3. Results

### 3.1. Preliminary Analyses

Prior to testing the hypothesized mediation model, descriptive statistics and bivariate analyses were conducted to examine the relationships among the main study variables. Preliminary analyses were also conducted to examine whether demographic characteristics (i.e., maternal age, maternal education, child age, and child sex) were associated with technoference and should therefore be included as covariates in the mediation model. Continuous demographic variables were examined using Pearson correlations, whereas maternal education, assessed as an ordinal variable, was examined using Spearman’s rank correlation. Differences according to child sex were evaluated using analysis of variance. Additionally, data distribution and regression assumptions were verified; all variables exhibited adequate normality, with skewness and kurtosis values falling well within the conventional limits of −1 and +1. Collinearity diagnostics revealed no multicollinearity issues (Tolerance values ranged from 0.703 to 0.965 and VIF values ranged from 1.036 to 1.422)**.** Further examination of regression assumptions across all equations included in the mediation model indicated no substantial violations of residual normality or homoscedasticity, based on visual inspection of normal P–P plots and standardized residual-versus-predicted plots. Cook’s distance and leverage values also indicated no influential observations (maximum Cook’s D values across equations = 0.048–0.058; maximum leverage values = 0.046–0.052).

Descriptive statistics and Pearson correlation coefficients are presented in Table 1 and Table 2. Loneliness was positively associated with nomophobia, social media addiction, and technoference. Nomophobia and social media addiction were correlated and both showed moderate positive associations with technoference. These findings indicated that the basic prerequisites for testing the mediation model were met, as significant associations were observed between the predictor, the mediators, and the outcome.

Regarding the role of sociodemographic variables, maternal age was negatively associated with technoference, nomophobia, and social media addiction, whereas it was not significantly associated with loneliness. Neither child age, child’s sex, nor maternal education was significantly associated with technoference; therefore, only maternal age was included as a covariate in subsequent analyses.

### 3.2. Parallel Multiple Mediation Analysis

To test the hypothesis that loneliness was indirectly associated with technoference through nomophobia and social media addiction, a parallel mediation analysis was performed using PROCESS version 4.2 (Model 4; Hayes, 2022) for SPSS (Table 3). Loneliness (UCLA) was entered as the independent variable, technoference (DISRUPT) as the dependent variable, nomophobia (NMP-Q) and social media addiction (BSMAS) as parallel mediators. Mothers’ age was entered as a covariate to control for its potential confounding effect. Confidence intervals (95%) for the indirect effects were generated using 5000 bootstrap samples.

Controlling for maternal age, loneliness significantly and positively predicted both nomophobia (β = 0.191; t = 3.605; *p* < 0.001) and social media addiction (β = 0.271; t = 5.277; *p* < 0.001).

When predicting technoference, both nomophobia (β = 0.318; t = 6.063; *p* < 0.001) and social media addiction (β = 0.310; t = 5.736; *p* < 0.001) were significant positive predictors, whereas the direct effect of loneliness was no longer significant (β = 0.029; t = 0.607; *p* = 0.544).

All three regression models were statistically significant. Loneliness and maternal age accounted for 5.1% of the variance in nomophobia (R^2^ = 0.051, F_(2, 340)_ = 9.11, *p* < 0.001) and 10.6% of the variance in social media addiction (R^2^ = 0.106, F_(2, 340)_ = 20.20, *p* < 0.001). The final model, including loneliness, both mediators, and maternal age, explained 30.6% of the variance in technoference (R^2^ = 0.306, F_(4, 338)_ = 37.22, *p* < 0.001).

With respect to covariate, maternal age was negatively associated with both nomophobia (β = −0.115; t = −2.17; *p* = 0.031), and social media addiction (β = −0.173; t = −3.37; *p* = 0.001), and was not significantly associated with technoference after controlling for loneliness, nomophobia, and social media addiction (β = −0.005; t = −0.116; *p* = 0.908).

As shown in Table 4, which presents the total, direct, and indirect effects of loneliness on technoference, the total effect of loneliness on technoference was significant (*B* = 0.473, *t* = 3.258, *p* = 0.001, 95% CI [0.187, 0.758]). The direct effect of loneliness on technoference became non-significant when controlling for the mediators. Bootstrap analyses indicated a significant total indirect effect (*B* = 0.394, 95% bootstrap CI [0.231, 0.572]). The specific indirect effect through nomophobia was significant (*B* = 0.165, 95% bootstrap CI [0.066, 0.290]), as was the specific indirect effect through social media addiction (*B* = 0.229, 95% bootstrap CI [0.118, 0.357]). The corresponding completely standardized indirect effects are also reported in Table 4. Thus, higher loneliness was associated with higher technoference indirectly through both higher nomophobia and higher social media addiction, even after accounting for maternal age.

**Figure 1:**
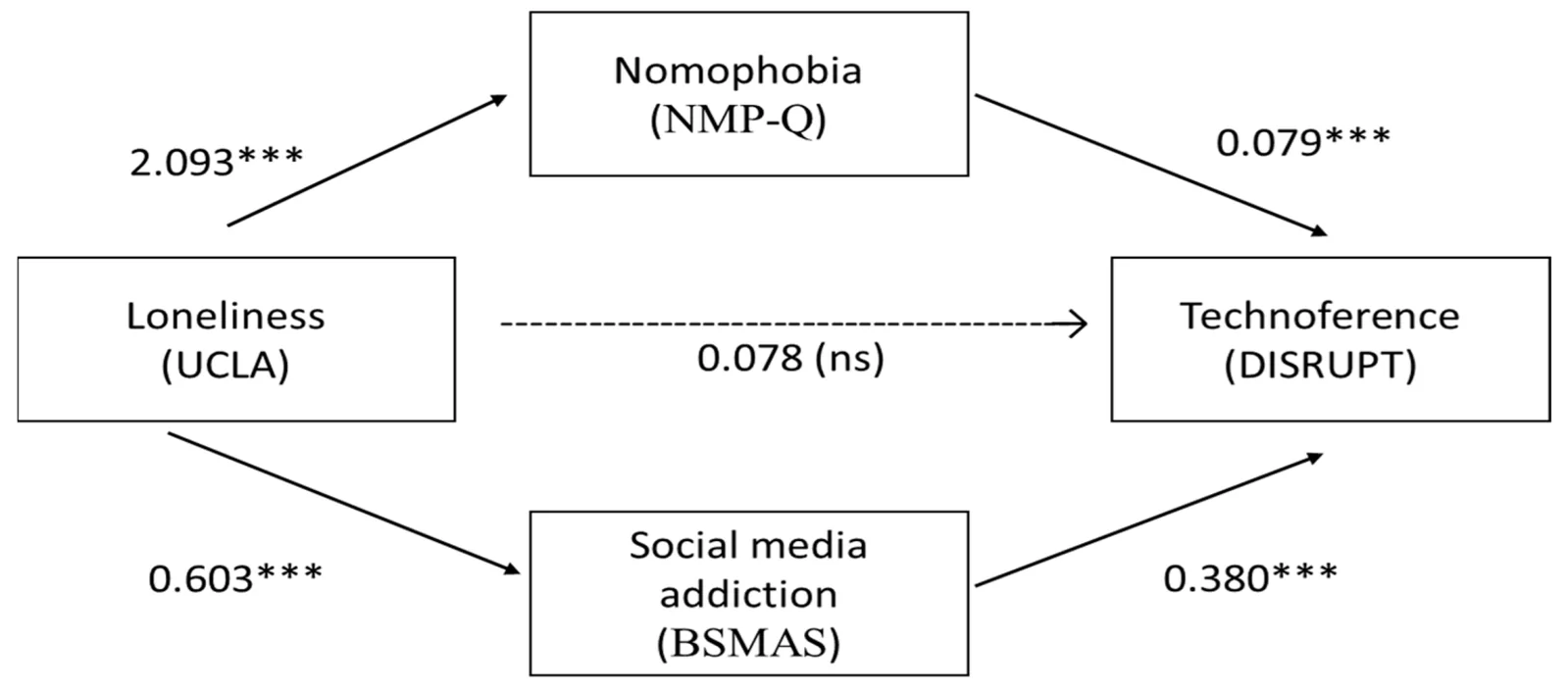
Mediation model: path coefficients for the relationship between UCLA, NMP_Q, BSMAS, DISRUPT. Covariate: Mother’s Age (included in the model). Note: *** *p* < 0.001, ns = not significant.

Overall, the findings support a parallel mediation model in which maternal loneliness is related to mother-child technoference through two partially distinct technology-related mechanisms: nomophobia and social media addiction. Because the direct effect of loneliness was no longer significant after the inclusion of the mediators, the association between loneliness and technoference appeared to be primarily indirect. The mediation model and its associated path coefficients are visually depicted in Figure 1.

The pairwise comparison of the specific indirect effects revealed no statistically significant difference between the two mediation pathways (contrast = −0.064, BootSE = 0.083, 95% bootstrap CI [−0.221, 0.103]). This indicates that nomophobia and social media addiction contributed similarly to explaining the association between loneliness and technoference.

## 4. Discussion

Previous research has demonstrated the negative impact of technoference on parents’ responsiveness and emotional availability, as well as on mother-infant co-regulation, an aspect that is particularly important during early childhood, when children rely heavily on the sensitivity of their primary caregivers to regulate their internal states and develop secure attachment relationships. However, considerably less attention has been devoted to understanding the psychological factors that may increase mothers’ vulnerability to technology-related disruptions during parent–child interactions. This study investigated the association between mothers’ perceptions of loneliness and technoference during interactions with children aged 0 to 6 years, examining the mediating roles of nomophobia and social media addiction.

Results confirmed our first hypothesis: mothers’ perception of loneliness was significantly and positively associated with both nomophobia and social media addiction. However, these associations were relatively modest, suggesting that loneliness is only one of several factors that may contribute to problematic digital technology use. The literature indicates that loneliness is an important yet underrecognized challenge during parenthood and is associated with reduced parental well-being and adverse consequences extending to children’s development (Nowland et al., 2024). Theoretical models of problematic Internet use propose that lonely individuals may cope with unmet relational needs through increased reliance on digital communication, which provide opportunities for social connection that may compensate for the lack of relationships in real life (Moretta & Buodo, 2020). Accordingly, digital devices may progressively acquire psychological significance that extends beyond their practical functions, providing individuals experiencing loneliness with a stable source of emotional reassurance and social connectedness. This psychological reliance may foster both problematic social media use and anxiety when access to the smartphone is restricted, two core features reflected by problematic social media use and nomophobia. It should be emphasized, however, that many studies on the relationship between loneliness and phone or Internet use have highlighted that this is a “chicken-and-egg question” (Moretta & Buodo, 2020), as it is not possible to identify with certainty the causal direction of the variables. Two complementary explanations have been proposed. Consistent with our hypothesis, individuals experiencing loneliness may increasingly turn to online activities to satisfy unmet social needs and regulate the distress associated with feeling socially disconnected. Conversely, persistent and maladaptive engagement with smartphones and online environments may gradually reduce involvement in face-to-face relationships, thereby reinforcing feelings of loneliness over time. To clarify this relationship, it would be necessary to conduct longitudinal research, given that most studies in this field, including ours, are cross-sectional in nature. Although the relationship is likely bidirectional, longitudinal evidence suggests that loneliness may exert a stronger influence on problematic technology use than vice versa (Hu & Xiang, 2024; Zhang et al., 2020). Nevertheless, reciprocal processes probably exist and deserve further investigation.

The second hypothesis of our study was that higher levels of nomophobia and social media addiction would be associated with higher levels of technoference during mother–child interactions. The results confirmed that nomophobia and social media addiction are factors associated with technoference during mother–child interactions, as noted in previous studies (Dolev-Cohen & Ricon, 2024; McDaniel & Radesky, 2018). Although conceptually distinct, nomophobia and social media addiction appear to represent complementary pathways through which problematic engagement with digital technologies may disrupt everyday mother-child interactions.

Our third hypothesis was that loneliness was indirectly associated with technoference through nomophobia and social media addiction. The literature suggests that lonely people are more likely to interact with others via smartphones or social media rather than through face-to-face communication, thereby escaping the social context of the real world (Zhan et al., 2022). Our results show that the relationship between perceived maternal loneliness and technoference in interactions with their children is only indirect, mediated by nomophobia and social media addiction. This pattern of full mediation is theoretically meaningful, as it suggests that loneliness per se may not directly associated with technoference. The association between loneliness and technoference may partly operate through problematic relationships with technology; however, the relatively modest associations observed for loneliness suggest that other factors are also likely to contribute to this link. This finding can be interpreted in light of the Compensatory Internet Use Theory (Kardefelt-Winther, 2014), which suggests that mothers may rely on digital communication to alleviate their sense of loneliness, which may in turn be associated with greater problematic smartphone and social media use. In this perspective, mothers who experience loneliness may develop a stronger psychological need to remain continuously connected to their smartphones, making it increasingly difficult to disengage from the device even during interactions with their children. Consequently, greater psychological dependence on digital technologies may increase mothers’ propensity to interrupt interactions with their children, reducing opportunities for sensitive, emotionally available exchanges and co-regulation.

In addition, pairwise comparison of the two indirect effects of nomophobia and social media addiction revealed that both play a significant role in the mediation model explaining the association between loneliness and technoference. They contributed similarly, with neither prevailing over the other.

This suggests that the link between loneliness and technoference does not stem from a single mechanism of problematic technology use, but rather from two dimensions that can be hypothesized as complementary: anxiety related to separation from one’s smartphone and problematic engagement with social media.

Overall, these findings suggest that technoference should be understood not simply as a consequence of smartphone use, but as the behavioral expression of underlying emotional and relational vulnerabilities. In this perspective, maternal loneliness emerges as a potential relational vulnerability, whereas nomophobia and social media addiction represent mechanisms through which this vulnerability may translate into everyday parenting behavior. At the same time, the relatively modest associations involving loneliness suggest that these processes likely reflect contributions from multiple individual, relational, and contextual factors. This interpretation is also consistent with the proportion of variance explained by the model (30.6%), indicating that a substantial share of variability in technoference is likely associated with other determinants not examined in this study.

### Limitations

The present study has several limitations that should be considered when interpreting the findings. First, the cross-sectional design precludes conclusions about the direction of the observed associations and does not allow causal inferences. Although the proposed model was theoretically grounded in the Compensatory Internet Use Theory, the relations among loneliness, nomophobia, social media addiction, and technoference may be bidirectional. For example, while loneliness may increase reliance on digital technologies, excessive engagement with smartphones and social media may, in turn, reinforce feelings of loneliness. Longitudinal studies are therefore needed to clarify the temporal ordering and reciprocal influences among these constructs.

Second, all variables were assessed through self-report measures, which may have introduced biases related to social desirability, and recall. Although Harman’s single-factor test did not indicate a substantial concern, the possibility of common method bias cannot be entirely ruled out. Future research would benefit from adopting multimethod approaches that combine self-report instruments with observational assessments of parent–child interactions and objective indicators of smartphone use (e.g., digital trace data or usage logs).

Third, although the present study focused on loneliness as a relational vulnerability underlying maternal technoference, other individual and contextual factors may also contribute to these processes. For example, parenting stress, parental self-efficacy, caregivers’ mental health, socioeconomic factors, and child characteristics such as temperament were not accounted for in the present analyses and may represent potential confounding factors, as they could be associated with both mothers’ relational and emotional experiences and their patterns of technology use. Therefore, the observed associations should be interpreted with caution. Future research should examine and control for these variables to provide a more comprehensive understanding of the factors associated with technoference in early parent–child interactions.

In addition, the use of a snowball sampling strategy may have introduced selection bias and limited the representativeness of the sample. Mothers reached through participants’ social networks who voluntarily completed an online questionnaire may systematically differ from the broader population of mothers of children aged 0–6 in sociodemographic characteristics, social connectedness, or familiarity with digital technologies. Consequently, the findings may not be fully generalizable to mothers with different backgrounds, levels of social connectedness, or familiarity with digital technologies. Future studies should use more diverse and representative sampling strategies to strengthen the external validity of these findings.

Finally, the study focused exclusively on mothers, reflecting their central caregiving role in early childhood. Although this choice aligns with the developmental focus of the study, it limits the generalizability of the findings to fathers and other caregivers. Future research should adopt a broader family perspective by including multiple caregivers, thereby clarifying how maternal and paternal digital behaviors jointly contribute to technoference and shape early parent–child interactions.

Addressing these limitations through longitudinal, multimethod, family-based research designs will provide a more comprehensive understanding of the psychological mechanisms underlying technoference and inform interventions aimed at supporting emotionally available parent–child relationships in the digital era.

## 5. Conclusions

The present study contributes to the growing literature on parental technoference by showing that maternal loneliness may be associated with technology-related disruptions in mother–child interactions through its associations with nomophobia and social media addiction. These findings suggest that technoference should not be understood simply as a consequence of digital device use, but as a relational phenomenon that may reflect mothers’ attempts to cope with unmet emotional and relational needs through digital technologies.

From a practical perspective, these findings indicate that interventions aimed at reducing technoference should extend beyond promoting healthier screen habits or limiting device use. Instead, they should address the emotional and relational needs that may underlie mothers’ engagement with digital technologies. Enhancing perceived social support and meaningful interpersonal connections may reduce reliance on digital technologies as a compensatory strategy and ultimately promote greater emotional availability during mother–child interactions.

Overall, these findings highlight the importance of considering the emotional and relational context in which technology use occurs. Supporting mothers’ psychological well-being and strengthening their relational resources may represent an important pathway toward healthier digital behaviors and emotionally available mother–child relationships in an increasingly digital society.

## Figures and Tables

**Table 1:** Descriptive statistics.

Variable	M	SD	Range	Sk	K
1. UCLA	5.26	1.80	3–9	0.40	−0.75
2. NMP-Q	47.23	19.78	20–116	0.93	0.52
3. BSMAS	12.34	3.99	6–27	0.79	0.70
4. DISRUPT	11.32	4.92	4–24	0.39	0.73

**Table 2:** Pearson correlations.

Variable	1	2	3	4	5	6
1. UCLA	—					
2. NMP-Q	0.194 **	—				
3. BSMAS	0.272 **	0.499 **	—			
4. DISRUPT	0.185 **	0.479 **	0.482 **	—		
5. Maternal age	−0.040	−0.121 *	−0.186 **	−0.113 *	—	
6. Child age	−0.001	−0.049	−0.061	−0.018	0.416 **	—

Note. — = correlations of variables with themselves are not reported. * Correlation is significant at the 0.05 level (two-tailed). ** Correlation is significant at the 0.01 level (two-tailed).

**Table 3:** Regression coefficients for the parallel mediation model.

Path	*B*	*SE*	*β*	*t*	*p*	95% CI Lower	95% CI Upper
UCLA→NMP-Q	2.093	0.581	0.191	3.605	<0.001	0.951	3.235
Maternal age→NMP-Q	−0.495	0.228	−0.115	−2.17	0.031	−0.945	−0.046
UCLA→BSMAS	0.603	0.114	0.271	5.277	<0.001	0.378	0.828
Maternal age→BSMAS	−0.152	0.045	−0.173	−3.37	0.001	−0.240	−0.063
Nomophobia→Disrupt	0.079	0.013	0.318	6.063	<0.001	0.053	0.105
Maternal age→Disrupt	−0.006	0.050	−0.005	−0.116	0.908	−0.103	0.092
BSMAS→Disrupt	0.380	0.066	0.310	5.736	<0.001	0.249	0.510
UCLA→Disrupt	0.078	0.129	0.029	0.607	0.544	−0.176	0.332

Note. *B* = unstandardized regression coefficient; *β* = standardized regression coefficient; CI = confidence interval.

**Table 4:** Total, direct, and indirect effects of loneliness on technoference.

	*B*	*SE*	*t*	*p*	95% CI Lower	95% CI Upper	CSIE [95% Boot CI]
Total effect	0.473	0.145	3.258	0.001	0.187	0.758	-
Direct effect	0.078	0.129	0.607	0.544	−0.176	0.332	-
Total indirect effect	0.394	0.086	-	-	0.231	0.572	0.145 [0.086, 0.206]
Indirect via nomophobia	0.165	0.058	-	-	0.066	0.290	0.061 [0.023, 0.106]
Indirect via social media addiction	0.229	0.061	-	-	0.118	0.357	0.084 [0.045, 0.129]
Contrast	−0.064	0.083	-	-	−0.221	0.103	-

Note. CI = confidence interval. CSIE = completely standardized indirect effect. For the total indirect effect, specific indirect effects, contrast and CSIEs, SEs and confidence intervals are based on 5000 bootstrap resamples.

## Data Availability

The data presented in this study are available on request from the corresponding author.
